# Validation of the medium and short version of CENSOPAS-COPSOQ: a psychometric study in the Peruvian population

**DOI:** 10.1186/s12889-022-13328-0

**Published:** 2022-05-07

**Authors:** Martha R. Lucero-Perez, Iselle Sabastizagal, Jonh Astete-Cornejo, Miguel Angel Burgos, David Villarreal-Zegarra, Salvador Moncada

**Affiliations:** 1 Instituto Nacional de Salud, Lima, Peru; 2 Universidad Peruana Cayetano Heredia, Lima, Peru; 3 Instituto Sindical de Trabajo Ambiente y Salud, Barcelona, Spain

**Keywords:** Psychometric, Occupational health, Occupational risks, Work, Peru

## Abstract

**Background:**

The presence of psychosocial risks at work are associated with mental and physical health issues in workers. The study aim was to adapt the COPSOQ-ISTAS21 (Spanish version of the Copenhagen Psychosocial Questionnaire and Union Institute of Work, Environment and Health) Medium-Version to the Peruvian context and to develop a Short-Version of the instrument.

**Method:**

Cross-sectional design study. The COPSOQ-ISTAS21 Medium Version was used. A confirmatory factor analysis was performed to determine the internal structure of each subdimension (first-order) and dimension (second-order) using the Robust Maximum Likelihood estimation method, and classic fit indices in the literature (CFI, SRMR, RMSEA). Internal consistency was evaluated using the alpha and omega coefficients. A short version was developed based on the items with the highest factorial load and that reduce the factorial complexity.

**Results:**

A total of 1707 participants were evaluated. In the confirmatory factor analysis, the goodness-of-fit indices for seventeen of the 20 one-dimensional models (subdimensions) were identified; two subdimensions could not be evaluated because they presented only two items. When conducting a multidimensional analysis, we identified that all second-order models presented optimal goodness-of-fit indices, except “psychological demands at work”. Finally, a short version of only 31 items was designed from the items with optimal fit indices.

**Conclusions:**

The new adapted versions of COPSOQ-ISTAS21 were renamed CENSOPAS-COPSOQ (National center of occupational health and environment protection for health -in Spanish- and Copenhagen Psychosocial Questionnaire). The CENSOPAS-COPSOQ is an instrument with sufficient evidence of validity and reliability in its medium and short version, which is why its use is recommended in Peruvian work centers to identify the evaluation and prevention of psychosocial risks at work in Peru.

**Supplementary Information:**

The online version contains supplementary material available at 10.1186/s12889-022-13328-0.

## Background

Globalization, technological advances, and changes in the working market have modified the behavior and health of workers, and the perception of the occupational risks to which they are exposed [1]. Occupational psychosocial risks are defined as aspects related to the design and management of work, as well as those related to the social and organizational sphere that has the potential to produce psychological or physical harm [2]. Occupational psychosocial risks are a complex concept, as they involve such work content, workload, work rhythm, work schedule, control, environment and team, organizational culture, function, interpersonal relationships at work, role in the organization, development career, work-at-home interference [3]. Exposure to these psychosocial occupational risks increases the risk of physical and mental health issues, including heart disease and stress [4–7]. Also, psychosocial occupational risks can have effects on multiple indicators of the work environment and organizational functioning such as absenteeism and decreased productivity [8].

Most of the reforms of working conditions and studies on working conditions have been carried out in high-income countries. [1]. Particularly, Peru a middle-income country has enacted some laws to improve working conditions and reduce occupational psychosocial risks. Among them, Law 29,783 on Occupational Health and Safety and its Regulations, where article N ° 30 indicates that employers must take into account the risks present in the workplace and specifically those related to the position or function of each employee [9]. Therefore, all Peruvian employers are responsible and have the obligatory for evaluating the risks to which each worker is exposed and are in charge of ensuring the development and implementation of accident prevention and protection standards, based on these risks [9].

To comply with the existing legislation and improve the work environment of workers, it is necessary to have valid and reliable tools to assess the psychosocial risks in the workplace. Currently, there are different instruments to evaluate the occupational psychosocial risks [10], such as the Job Content Questionnaire (JCQ) [11]; Effort Reward Imbalance (ERI) Questionnaire [12]; COPSOQ (Copenhagen Psychosocial Questionnaire) [13]; General Nordic Questionnaire on Psychological and Social Factors at Work (QPS Nordic) [14].

Despite the variety of instruments for assessing occupational psychosocial risks, the COPSOQ has characteristics that differentiate it from most instruments [11], especially the ERI [12] and the JCQ [11]. Four advantages of the COPSOQ can be pointed out, compared to the rest of the instruments. First, it is not based solely on classical theoretical models such as ERI or JCQ, but links occupational psychosocial risks, the work environment and the effects on the worker's mental health; for this reason, the COPSOQ psychosocial risk model is not only a predictor of work stress [15]. Second, the COPSPQ has an epidemiological basis, which defines units of analysis in three sections (improve, maintain and promote) and allows a measure of assessment and intervention of the workplace [16]. Third, it incorporates indicators for specific sectors and occupations, which allows better specifying the levels of exposure to occupational psychosocial risk [16]. Finally, it is adaptable to all types of workplaces, since it was developed in the analysis and prevention of occupational hazards [16].

The COPSOQ-ISTAS-21 (Spanish version of the Copenhagen Psychosocial Questionnaire) has three types of versions (long, medium and short version) [17]. However, our study will only use two of them, which are the most commonly used (medium and short version). The medium version of the instrument has 69 items, and this version is used in companies with more than 25 workers. While the short version includes the most representative items of the medium version of the COPSOQ-ISTAS-21 and is used for companies with 25 or fewer workers. As has been shown, there is information that supports the usefulness of the COPSOQ-ISTAS-21 to measure the psychosocial risks at work in the workplace. For this reason, our objective is to know the validity and reliability indicators of the COPSOQ-ISTAS-21 in the context of Peruvian companies and to prepare a short version for companies with 25 to fewer workers. It should be noted that it is important that this study be carried out in formal Peruvian companies since there is currently legislation that requires the development of occupational evaluations that assess psychosocial risks in the work context; however, there are no validations of the COPSOQ-ISTAS-21 within the Peruvian context. Therefore, this study will allow a first approximation of the measurement properties in this particular group.

## Methods

### Design

This is a cross-sectional and psychometric study. The study was conducted from July 01, 2016 to February 28, 2017. Data were collected in different cities in Peru from companies with 25 or more workers. The companies where the data were collected were formal and came from six economic activities (Extractive, Manufacturing, Construction, Services, Transportation, and Communications).

### Participants

The sampling was non- randomized. The sample of this study consisted of workers from a list of companies registered at the National Superintendency of Tax Administration (SUNAT) with more than 25 workers. Workers over 14 years of age, with more than a month of service at the time of application of the questionnaire, literate, and who had signed the informed consent were included. Likewise, workers in the process of dismissal or immersed in administrative processes were excluded. The participants worked at six of the most important economic activities in Peru.

Through a sample size calculation with a 95% confidence level, a minimum size of 1604 workers were obtained. However, in the questionnaire application stage, a sample of 1707 workers were obtained; a fact that does not affect the validity of the study due to the nature of the research design.

### Instrument

The COPSOQ is a Likert-type instrument of Danish origin, which was translated, adapted, and validated in Spain: being renamed COPSOQ-ISTAS-21, which assesses exposure to psychosocial risk factors at work considering working conditions in which this is done [17]. With response options ranging from always (5 points) to never (1 point). This instrument has 3 versions: a long (research), a medium (companies with more than 25 workers), and a short (companies with less than 25 workers). In this instrument, standardized scores can be obtained in a range from 0 to 100, in addition to grouping workers into terciles (green, yellow, red) classified as "most favorable for health", "intermediate" and "most unfavorable for health” respectively.

### Procedures

The tests were applied to workers in workplaces with more than 25 workers according to the different economic activities and regions of Peru, by a team of psychologists trained and supervised by the researchers. Also, all participants were given an informed consent that had to be signed voluntarily, if they wished to participate in the Study. It should be noted that the questionnaire was previously adapted and agreed upon to the Peruvian reality by a work team made up of representatives of employers, workers, and researchers.

On the other hand, to perform the confirmatory factor analysis (CFA), correlation and reliability analysis, the statistical program R Project was used [18].

People who were included in the study had to read and sign the informed consent to voluntarily participate in the study. Likewise, participation was anonymous, and no information was included in the database that would allow them to be identified. Therefore, this study does not represent an ethical risk. The protocol has been approved by the Institutional Committee for Research Ethics of the National Institute of Health (No. RD 563–2015-OEI-OGITTOPE / INS). Necessary ethical care was maintained following the guidelines of the Declaration of Helsinki.

Before the execution of the field study, a process of cultural adaptation of the instrument was carried out by a linguist. This adapted version underwent a content validation process through working groups made up of 60 experts, including the researchers of this study. The working groups were made up of representatives of the business sector (National Confederation of Private Entrepreneurial Institutions-CONFIEP, and others), representatives of workers' unions, representatives of universities that train in occupational health (Universidad Nacional Mayor de San Marcos, and Universidad Peruana Cayetano Heredia), and representatives of state entities linked to occupational health (Ministry of Health, Ministry of Labor and Employment Promotion, EsSalud, CENSOPAS-INS).

The working groups evaluated the clarity and coherence of the items, as well as the relevance of their inclusion in the dimensions of the construct. This led to the modification of the wording of items based on the theoretical analysis, and the final version was validated by all members.

### Analysis of data

#### Confirmatory factor analysis

The estimator used was weighted least squares means and variance adjusted (WLSMV), and polychoric correlation matrices because they better fit the categorical-ordinal nature of the items. Twenty one-dimensional models were evaluated (all the items in a model evaluate a single dimension, i.e. they can be added together to obtain an overall score), evaluating the factorial structure of each group of items according to the corresponding dimensions described in the COPSOQ-ISTAS 21. Furthermore, the factorial structures of 6 s-order models (or multidimensional models) that encompass the 20 aforementioned subdimensions were evaluated, according to their theoretical link with 6 COPSOQ-ISTAS 21 psychosocial risk constructs (items can be aggregated into an overall dimension and into sub-dimensions). All these analyzes correspond to the average version of the COPSOQ-ISTAS-21 instrument (69 items). To conduct this analysis, the weighted least squares means and variance adjusted (WLSMV) was used. The assumptions of the model that WLSMV is that the data are ordinal and do not require compliance with the non-normality of the data [19].

The different models of the COPSOQ-ISTAS-21 medium version was evaluated based on two steps. First, different indicators were used to determine the fit of each of the models (one-dimensional and second-order). The comparative fit index (CFI) was used, whose appropriate values are taken ≥ 0.90 [20]. Likewise, the Standardized Root Mean-Square (SRMR) and the Root Mean Square Error of Approximation (RMSEA) with a confidence interval of 90% were used, which categorize as adequate value < 0.08 [21]. Second, all models must have at least three items to be evaluated, since it is the minimum number of items that allows an instrument to be stable [22].

#### Reliability

Based on the models identified in the factorial analysis, the reliability of the CENSOPAS-COPSOQ instrument was evaluated, medium version, through the analysis of internal consistency, reporting values of classical alpha (α) and categorical omega coefficient (ω) [23]. In both coefficients, the optimal values are > 0.70.

#### Development of a short version

Based on the evaluated models of the CENSOPAS-COPSOQ medium version, we sought to develop a short version aimed at assessing psychosocial risks in public or private workplaces with fewer than 25 workers. To this end, the strategy was to select the most representative items of each of the twenty subdimensions and gradually eliminate the items that contributed low variance (low factor loadings) or introduced factorial complexity to the model.

Four steps were proposed to develop this short version. First, the number of items was reduced. The second-order models were collapsed into only six unidimensional models based on the original 20 subdimensions (69 items). The 20 subdimensions were not taken into account in this step, as they would not be stable by themselves. Second, once the items were collapsed into six unidimensional models, items were sequentially removed from each model until adequate fit indices (CFI ≥ 0.90; RMSEA and SRMR < 0.08) were achieved. During item elimination, we tried to keep those items that were more representative (with higher factor loadings) and with lower complexity (without correlation errors). Third, within the six unidimensional models of the short version of CENSOPAS-COPSOQ, it was ensured that there were always between one and two items from each of the 20 original subdimensions. This criterion was adopted to avoid eliminating items unnecessarily and ending up partially evaluating the construct. An exception to this criterion was considered to be the model of the dimension of control over work since the minimum number of elements for a dimension to be stable is three elements [22]. Fourth, item removal was stopped when the optimal fit indices were reached or when there was at least one item from each of the 20 subdimensions within the model.

#### Convergent validity

A correlation analysis was performed between the dimensions of the short version of CENSOPAS-COPSOQ with the second-order dimensions and sub-dimensions of the medium version of CENSOPAS-COPSOQ (convergent validity). It is expected that the more the dimensions were related more similar their scores will be high correlation values would suggest that there is no missing information between the short and medium versions. The values of the spearman correlation are specified as very high (*r* > 0.9) high (*r* > 0.7), moderate (*r* > 0.5) and low (*r* > 0.3) correlations [24].

## Results

### Participants

The questionnaire was applied to a total of 1,707 workers, distributed throughout the country in three major geographic regions: Coast (Lima, Ica, La Libertad, and Piura, with a proportion of 35%), Highlands (Arequipa, Huánuco, Junín, Pasco, Huancavelica, and Cuzco, representing 33.4%) and Rainforest (San Martín, Ucayali, Madre de Dios, and Loreto, representing 31.6% of the sample).

The highest proportion of the population were males (61%), and aged under 31 (42%), and only a minority were over 45 years of age (17.3%). Regarding their education, 29.6% of the sample had completed university education, followed by those with a complete technical and complete secondary education (19.2% and 18% respectively), and only 1.5% had incomplete primary education.

### One-dimensional models

Seventeen of the 20 one-dimensional models presented adequate fit indices (see Table 1). It should be noted that eight of the seventeen one-dimensional models evaluated presented high RMSEA values.

**Table 1:** Adjustment indices of the CENSOPAS-COPSOQ one-dimensional models

No	Sub-dimensions	N ° items	*X*^*2*^	*df*	CFI	SRMR	RMSEA [90% CI]	α	ω	λ range
1	Quantitative requirements	4	6.5*	2	0.999	0.013	0.036 [0.007–0.069]	0.67	0.71	0.81–0.69
2	Pace of work	3	0	0	1	0	0	0.59	0.62	0.83–0.40
3	Emotional demands	4	71.9*	2	0.978	0.037	0.143 [0.116–0.173]	0.74	0.74	0.80–0.60
4	Demands to hide emotions	4	143.4*	2	0.916	0.07	0.204 [0.176–0.233]	0.63	0.65	0.78–0.42
5	Double presence	4	121.3*	2	0.993	0.027	0.187 [0.160–0.216]	0.88	0.88	0.92–0.74
6	Influence	4	20.6*	2	0.999	0.011	0.074 [0.047–0.104]	0.83	0.84	0.94–0.60
7	Development possibilities	4	39.5*	2	0.992	0.025	0.105 [0.078–0.135]	0.74	0.75	0.85–0.58
8	Sense of work	3	0	0	1	0	0	0.85	0.85	0.92–0.80
9	Social support from peers	3	0	0	1	0	0	0.78	0.78	0.85–0.73
10	Social support from superiors	3	0	0	1	0	0	0.90	0.90	0.96–0.86
11	Leadership quality	4	96.3*	2	0.998	0.015	0.166 [0.139–0.195]	0.92	0.92	0.96–0.81
12	Group sentiment	3	0	0	1	0	0	0.86	0.86	0.90–0.84
13	Predictability	2	-	-	-	-	-	-	-	-
14	Role clarity	4	190.9*	2	0.953	0.06	0.235 [0.208–0.264]	0.74	0.75	0.79–0.66
15	Role conflict	4	38.8*	2	0.99	0.029	0.104 [0.077–0.134]	0.68	0.70	0.91–0.41
16	Recognition	3	0	0	1	0	0	0.84	0.85	0.90–0.75
17	Job insecurity	2	-	-	-	-	-	-	-	-
18	Insecurity about working conditions	4	38.9*	2	0.996	0.02	0.104 [0.077–0.134]	0.84	0.84	0.87–0.75
19	Justice	4	15.4*	2	0.998	0.012	0.063 [0.036–0.094]	0.80	0.80	0.87–0.68
20	Vertical trust	3	0	0	1	0	0	0.78	0.79	0.87–0.63

^*^Values are significant (*p* < 0.05). X^2^ = Chi-squared. df = Degrees of freedom. CFI = Comparative fit index. RMSEA = Root mean square error of approximation. SRMR = Standardized root mean square residual. α = alpha coefficient of internal consistency. ω = omega coefficient of internal consistency. λ range = Range of factor loadings

Sixteen of the 20 one-dimensional models presented optimal values of internal consistency (reliability). However, the model of "work rhythm" and "demand to hide emotions" showed slightly low internal consistency values (see Table 1).

The dimensions of "predictability" and "insecurity about employment" did not present adjustment indices or internal consistency values since a minimum of 3 items per dimension is required. Therefore, these two one-dimensional models were not considered for this analysis. This does not affect the validity of these two dimensions since they were evaluated in the following stages of the analysis.

### Multidimensional models

It was identified that all second-order models presented adequate adjustment indices, except the model of "psychological demands at work", which presented low adjustment indices. The second-order dimensions of Work-family conflict, Control over work, Social support and quality of leadership, Work compensation, and Social capital presented adequate evidence of internal structure validity.

It was identified that all second-order models presented optimal internal consistency values in all cases (see Table 2). The selected models are graphically represented in Supplementary Figs. 1, 2, 3, 4, 5, 6. When analyzing the factor loadings and latent variables, the second-order model "Control over work" presents an overestimation for the subdimension "development possibilities", since it would overexplain the total variance of the construct (see Supplementary Figs. 3). On the other hand, the second-order model "Social support and leadership quality" present an underestimation in the subdimension "role conflict", since it explains only 5% of the total construct (see Supplementary Figs. 4). Likewise, the second-order model "Work compensation" presents two of its three subdimensions that explain little of the construct, reaching values between 3 and 4% (see Supplementary Figs. 5).

**Table 2:** CENSOPAS-COPSOQ second-order models adjustment indices

Dimensions	N ° items	*X*^*2*^	*df*	CFI	SRMR	RMSEA	Ω _hierarchical_	λ range
Psychological demands at work	15	3020.0*	86	0.826	0.091	0.142 [0.137–0.146]	0.94	0.99–0.41
Work-family conflict	4	121.3*	2	0.993	0.027	0.187 [0.160–0.216]	0.88 **	0.92–0.74
Control over work	11	587.4*	41	0.980	0.051	0.088 [0.082–0.095]	0.74	0.94–0.64
Social support and quality of leadership	23	3419.3*	223	0.955	0.071	0.092 [0.089–0.094]	0.87	0.95–0.35
Work compensation	9	431.6*	24	0.984	0.044	0.100 [0.092–0.108]	0.87	0.90–0.73
Social capital	7	665.2*	12	0.963	0.075	0.179 [0.167–0.190]	0.89	0.84–0.63

Medium version = 69 items. * Values are significant (*p* < 0.05). ** Omega of the first order. X^2^ = Chi-squared. df = Degrees of freedom. CFI = Comparative fit index. RMSEA = Root mean square error of approximation. SRMR = Standardized root mean square residual. Ω = hierarchical omega coefficient. λ range = Range of factor loadings

The new adapted versions of COPSOQ-ISTAS21 were renamed CENSOPAS-COPSOQ. The name change is justified due to the adaptation of the new items, evaluation of their measurement properties, and subsequent development of a short version.

### A short version of the instrument

To develop the short version of the instrument, items of each first-order dimension were eliminated sequentially (supplementary table 1), until the most stable versions of the instrument were left. The most stable versions, with better fit indices and internal consistency, are presented in Table 3. Although all the models of the short version presented optimal fit indices, the seven-item model of “social support and leadership quality” has high RMSEA values. These values improve when the items corresponding to "peer social support" (item 28a) and "group feeling" (item 28e) are related, reaching optimal values (gl = 13; CFI = 0.98; SRMR = 0.03; RMSEA = 0.08).

**Table 3:** Adjustment indices of the short version of the CENSOPAS-COPSOQ

Dimensions	N original items	Reduced version	*X*^*2*^	*df*	CFI	SRMR	RMSEA	α	Ω	λ range
Psychological demands at work	15	7	184.8*	14	0.975	0.4	0.085 [0.074–0.096]	0.79	0.80	0.92–0.77
Work-family conflict	4	3	0	0	1	0	0.000 [0.000–0.000]	0.87	0.87	0.89–0.37
Control over work	11	5	56.0*	5	0.993	0.026	0.077 [0.060–0.096]	0.73	0.74	0.78–0.24
Social support and quality of leadership	23	7	287.9*	14	0.962	0.046	0.107 [0.097–0.118]	0.74	0.75	0.89–0.19
Work compensation	9	5	24.5*	5	0.999	0.018	0.048 [0.030–0.068]	0.81	0.85	0.86–0.66
Social capital	7	4	16.1*	2	0.998	0.011	0.064 [0.038–0.095]	0.81	0.82	0.80–0.36

Medium version = 69 items. Short version = 31 items. * Values are significant (*p* < 0.05). X^2^ = Chi-squared. df = Degrees of freedom. CFI = Comparative fit index. RMSEA = Root mean square error of approximation. SRMR = Standardized root mean square residual. α = alpha coefficient of internal consistency. ω = omega coefficient of internal consistency. λ range = Range of factor loadings

It should be noted that all the models of the short version have optimal reliability values (see Table 3). The selected models are graphically represented in Supplementary Figs. 7, 8, 9, 10, 11, 12.

The items of the medium-version and short-version in English can be found in supplementary table 2.

In the analysis of the short version of the CENSOPAS-COPSOQ, four items were identified as having factor loadings lower than 0.40 (istas26m, istas30a, istas27c, istas25j). Although these items have low factor loadings compared to the rest, we consider that they are theoretically relevant, since otherwise the construct would be partially evaluated, since items of the twenty original dimensions would not be considered.

The convergent validity of the short version of the CENSOPAS-COPSOQ instrument with the medium version was evaluated in supplementary table 3. A high correlation (r > 0.90) was identified between the dimensions of the short version with the second-order dimensions of the medium version (psychological demands at work, work-family conflict, control over work, social support and quality of leadership, work compensation, and social capital).

## Discussion

### Main conclusions and meaning of the results

The CENSOPAS-COPSOQ presented evidence of validity and reliability for the medium version and the short version, therefore its use could be recommended in the Peruvian population to assess occupational psychosocial risks. The goodness of fit indices supports the evidence of the validity by internal structure, while the internal consistency coefficients of alpha and omega support the reliability of the measurement for both versions. In addition, it was identified that the medium and short versions of CENSOPAS-COPSOQ are closely related so it is unlikely that information would be lost in the use of a shorter version.

However, the "psychological demands at work" dimension in the medium version presents an inadequate fit, but good levels of reliability. Therefore, the results of this dimension in particular should be taken with caution in the medium version. Also, some models have adequate CFI values but inadequate RMSEA values. Since we have few degrees of freedom and the sample is small compared to the number of parameters used, it is advisable to consider only the CFI values [25, 26]. Therefore, we consider that the goodness-of-fit index values are adequate, suggesting an adequate fit. In addition, some subdimensions of the CENSOPAS-COPSOQ were identified as having overestimated values and others as underestimated since they explain a low proportion of the total variance of the construct. This is to be expected in multifactorial instruments that evaluate a variable as complex as psychosocial risks. Therefore, although there are some problematic subdimensions within the instrument, this does not detract from the overall assessment of the instrument.

### Contrasting the findings with the existing literature

Different studies evaluated the measurement properties of the COPSOQ, using heterogeneous analysis methods and providing results that included different factorial solutions both in the number of items and in the number of dimensions.

Two studies were identified that present inadequate analysis methods, which suggested unstable factor solutions. In Spain, the validity of the COPSOQ-ISTAS-21 in workers was analyzed through a factor analysis with varimax rotation, and the internal consistency of the model was evaluated through the Alpha coefficient [27]. However, this rotation method used was not adequate since it assumed that the items and dimensions are independent, which does not happen within the psychological variables since they are closely related to each other [28], even more in an instrument with such factorial complexity (i.e., with a large number of dimensions and subdimensions). In this Spanish study, 27 subdimensions were found, possibly due to the use of an analysis method that does not conform to the nature of the variables (categorical-ordinal). The short version of the instrument they presented had 38 items [27], which represents 7 more items than the one presented in our study. On the other hand, a study carried out in Brazil validated the average version of the COPSOQ-ISTAS-21 II in university workers [29]. In this study, the authors state that they used confirmatory factor analysis, exploratory factor analysis, and reliability analysis by internal consistency [29], managing to identify a factorial model composed of 21 subdimensions that grouped 70 items. However, in the analysis plan of their article, they point out that they used analysis by principal components, varimax rotation, and the number of dimensions was determined with the eigenvalue. This method of analysis is not recommended as it is highly subjective and can overestimate the number of dimensions [28].

Two other studies identified four-dimensional models, which diverges from our findings where six dimensions were identified in the Peruvian population. A Persian study by COPSOQ evaluated the short version of the instrument [30], finding through confirmatory factor analysis a total of 4 dimensions (32 items): quality of leadership, social support from supervisors, rewards, justice and respect, trust, and predictability (dimension 1), self-rated health, burnout, stress, work-family conflict and emotional demands (dimension 2), the meaning of work, commitment to the workplace, influence at work and role clarity (dimension 3), and offensive behavior (dimension 4). Unlike the CENSOPAS-COPSOQ (our study), the Persian study by Aminian et al., collapses dimensions to achieve a more stable factorial solution. However, conceptually they maintain the same indicators as our study. On the other hand, a study validated the French version of the COPSOQ based on the short version in the Danish language [31]. Through exploratory factor analysis, only 4 dimensions were presented ("interpersonal relationships and leadership", "influence and development", "tension", and "demands") with 32 items. This version is similar in the number of items that are proposed in our study for the short version.

Two other studies argued that a five-dimensional version is more stable compared to other factorial solutions. One of them adapted the medium version of the COPSOQ to Persian and concluded that the items were grouped into 20 subdimensions [32], just like in our model. However, in this study, these subdimensions were grouped into only 5 dimensions (Type of production and tasks, Organization and content of work, Interpersonal relationships and leadership, Work-individual interaction, and Health and well-being) [32]. Likewise, a study that evaluated the validity of the COPSOQ in professional drivers in Spain also reported a better factor solution with 5 dimensions (“Demands”, “Influence and development”, “Interpersonal relationships and leadership”, “Job Insecurity” and “Strain”)[33]. These two studies present similar results to each other and by what our research identified since their dimensions are very similar to those proposed in our study.

A Chilean study carried out a validity and reliability analysis for the short version of the COPSOQ-ISTAS-21, which they called SUSESO-ISTAS21 [34]. This study proposed a factorial structure of 5 dimensions ("Psychological demands"; "work and skills development"; "social support in the company and quality of leadership"; "compensation" and "double presence") with a total of 20 items. However, this structure composed of 5 dimensions did not present adequate adjustment indices (CFI = 0.795; TLI = 0.762; RMSEA = 0.080) [21]. Despite presenting problems in its validity, it did achieve adequate internal consistency coefficients (α > 0.70) [23]. Therefore, although the instrument presents evidence of reliability, its factorial structure is unstable, and a correlated five-dimensional solution might not be the most appropriate.

The COPSOQ presents several studies that prove its convergent validity; however, because the versions of the instrument used are very heterogeneous, a direct comparison is not possible. Our study identified a very high relationship between the medium and short version of the instrument, which suggests that the instrument does not lose information by eliminating items. However, since the medium version has the same items as the short version, the data would be interdependent. Despite this, when evaluating the relationship with the unidimensional models, a moderate correlation is still identified, so we consider that no information would be lost between one version and the other.

The COPSOQ studies have presented heterogeneous factor structures and each one has obtained different dimensions and different numbers of items, according to the characteristics of their populations. Also, measurement properties have been evaluated in various ways such as exploratory and confirmatory factor analyses, and some studies have used poorly recommended analytical methods that could lead to unstable results [27, 29]. It should be noted that our study provides the use of second-order models, which allows a global score to be obtained by dimension and considers the 20 sub-dimensions within its structure. Therefore, we consider that our study provides a new approach to the available evidence from COPSOQ-ISTAS21 and is renamed CENSOPAS-COPSOQ.

The evidence presented corresponds to the second edition of COPSOQ; however, it should be noted that the third edition of COPSOQ was published in December 2019 [35]. The third edition adds a set of mandatory core items that must be included in all versions of the instrument (short, medium, or long), regardless of the country or context that adapts the scale. This will allow future studies conducted with the third edition of COPSOQ to have a constant set of items in all versions, which will allow comparisons to be made between countries and versions. This represents an important advance for the measurement of psychosocial risks because it will allow knowing the most appropriate items to the context but respecting a set of mandatory core items.

### Strengths and limitations

Our CENSOPAS-COPSOQ study had a representative sample of workers from different economic activities and regions of Peru, allowing the representativeness of workers with different characteristics. However, the present study is not without limitations. First, evaluations using bifactor models, or ESEM could not be carried out, since their sample size was insufficient to make the models converge; so, there could be other factorial solutions that could be more stable using these models. Second, CENSOPAS-COPSOQ collects the information on the psychosocial risks at work perceived by the worker, however, an identification of the psychosocial risk at the workplace was not carried out, to corroborate the perception of the worker. Third, it was not possible to apply other instruments that assess occupational psychosocial risks, which allows other validity evidence to be presented as evidence of relationship with other variables of the CENSOPAS-COPSOQ (discriminant validity). Fourth, an analysis of invariance by sex, economic sector and the natural region was attempted, but was not possible because the assumptions of the analysis were not met (not all participants checked all the response options) [36].

### Implications for public health and decision-making

The COPSOQ is an instrument used in different contexts and countries to assess psychosocial risks at work. Therefore, it is a valid and reliable tool that would allow directing public policies and periodic evaluations for work centers of 25 people or less with the short version (31 items), as well as for work centers with more than 25 workers with the medium version (69 items). Other countries have used COPSOQ to design and evaluate their labor policies [10], therefore, this instrument can be used in Peru as a tool to direct decision-making in occupational and occupational health.

The CENSOPAS-COPSOQ could be used to evaluate the effect of organizational or labor interventions since it would allow seeing if the safety and risk management strategies applied in the workplace have a positive or protective effect on the health of the workers, by carrying out evaluations before and after the interventions. Therefore, the CENSOPAS-COPSOQ joins other instruments adopted in the Peruvian context to evaluate labor aspects in public health [37, 38].

## Conclusions

The CENSOPAS-COPSOQ is an instrument with sufficient evidence of validity and reliability in its medium and short version, so its use is recommended in Peruvian work centers to identify, to assess and prevent psychosocial occupational risks.

## Supplementary Information

**Additional file 1:** **Supplementary figure 1.** Dimension Psychological demands atwork for the medium version. **Supplementary figure 2.** Family workconflict dimension for the medium version. **Supplementary figure 3.** Dimension Control overwork for the medium version. **Supplementary figure 4.** Socialsupport and leadership quality dimension for the medium version. **Supplementary figure 5.** Workcompensation dimension for the medium version. **Supplementary figure 6.** Capital stock dimensionfor the medium version. **Supplementary figure 7.** Family workconflict dimension for the short version. **Supplementary figure 8.** DimensionControl over work for the short version. **Supplementaryfigure 9.** Social support and leadership quality dimension for the short version.** Supplementary figure 10.** Workcompensation dimension for the short version. **Supplementary figure 11.** Capital stock dimensionfor the short version.** Supplementary figure 12. **DimensionPsychological demands at work for the short version. **Supplementary table 1.** Elimination of each ofthe possible models of the short version. **Supplementary table 2.** Items in English fromCENSOPAS-COPSOQ. **SupplementaryTable 3.** Correlation between the dimensions of the short version(columns) with the second-order dimensions and subdimensions of the mediumversion (rows).

## Data Availability

The database is available at https://doi.org/10.6084/m9.figshare.14138660
