# Supplementary material for: Validation of the medium and short version of CENSOPAS-COPSOQ: a psychometric study in the Peruvian population
Source: BMC Public Health. 2022 May 7;22:910. doi: 10.1186/s12889-022-13328-0 (PMC9077908; doi:10.1186/s12889-022-13328-0)
Supplement: Supplementary file 1 — Additional file 1: Supplementary figure 1. Dimension Psychological demands atwork for the medium version. Supplementary figure 2. Family workconflict dimension for the medium version. Supplementary figure 3. Dimension Control overwork for the medium version. Supplementary figure 4. Socialsupport and leadership quality dimension for the medium version. Supplementary figure 5. Workcompensation dimension for the medium version. Supplementary figure 6. Capital stock dimensionfor the medium version. Supplementary figure 7. Family workconflict dimension for the short version. Supplementary figure 8. DimensionControl over work for the short version. Supplementaryfigure 9. Social support and leadership quality dimension for the short version. Supplementary figure 10. Workcompensation dimension for the short version. Supplementary figure 11. Capital stock dimensionfor the short version. Supplementary figure 12. DimensionPsychological demands at work for the short version. Supplementary table 1. Elimination of each ofthe possible models of the short version. Supplementary table 2. Items in English fromCENSOPAS-COPSOQ. SupplementaryTable 3. Correlation between the dimensions of the short version(columns) with the second-order dimensions and subdimensions of the mediumversion (rows). [file 12889_2022_13328_MOESM1_ESM.docx]

Supplementary figure 1. Dimension Psychological demands at work for the medium version.


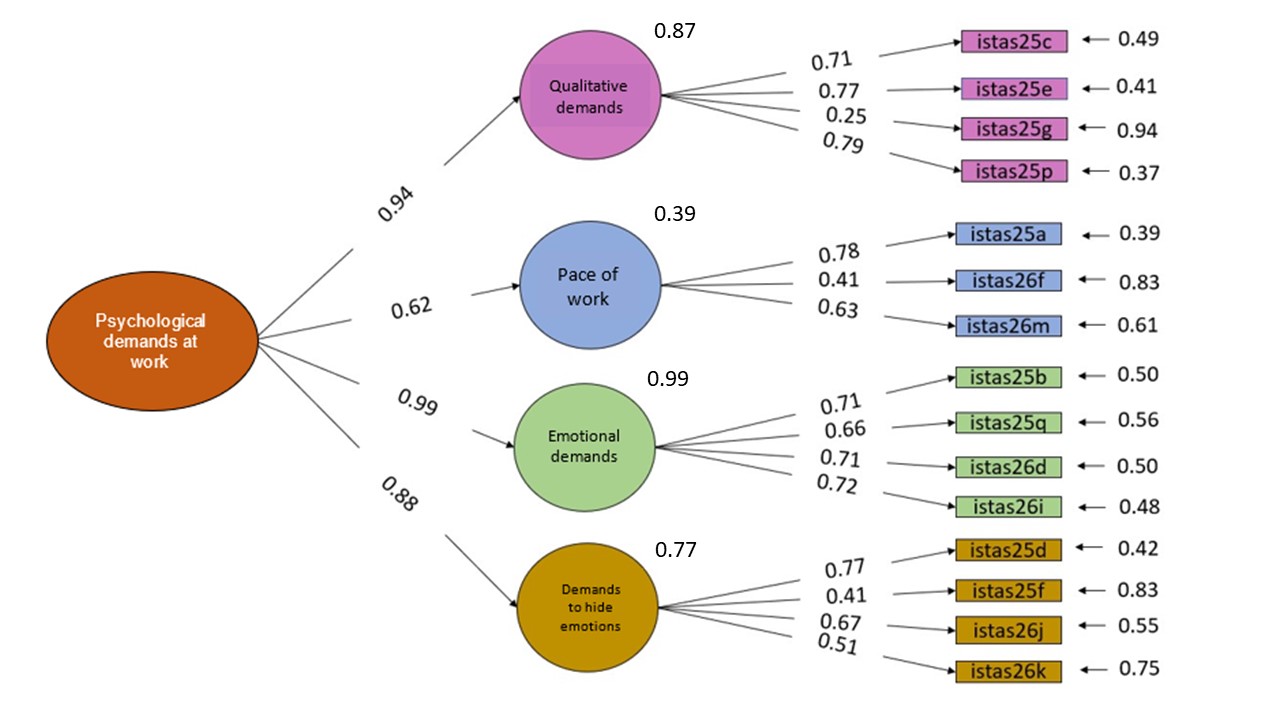


Supplementary figure 2. Family work conflict dimension for the medium version.


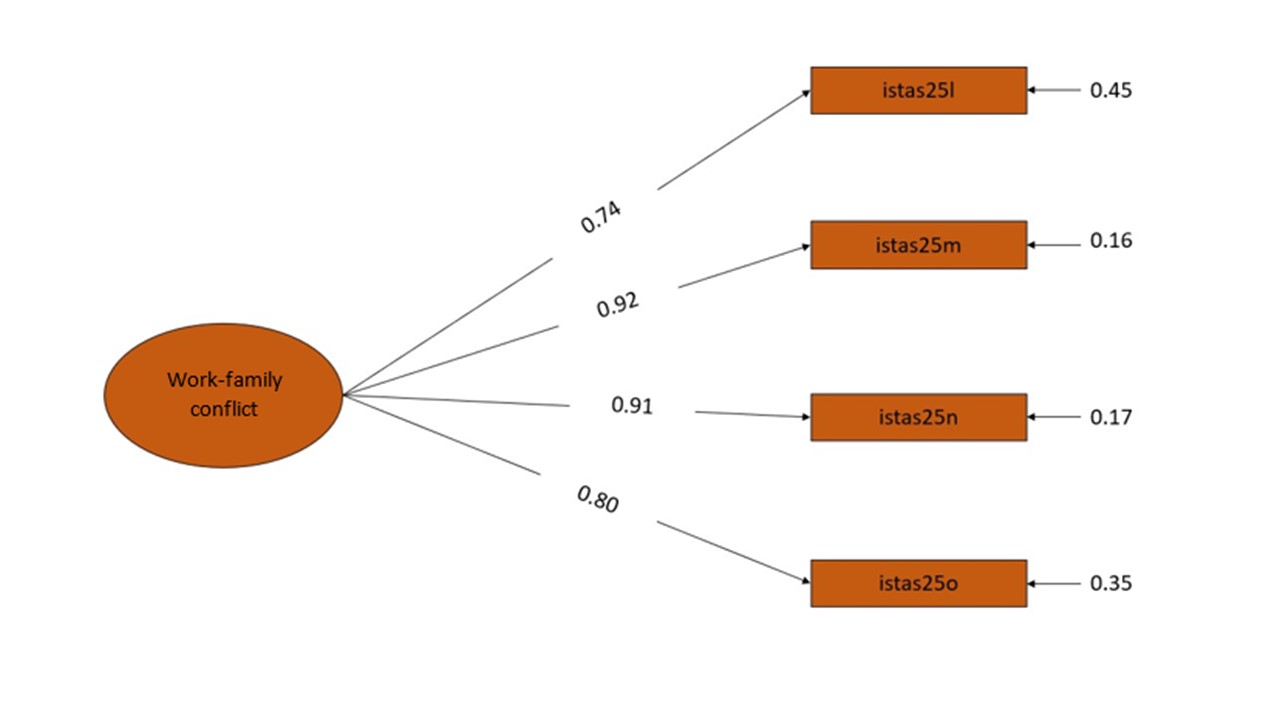


Supplementary figure 3. Dimension Control over work for the medium version.


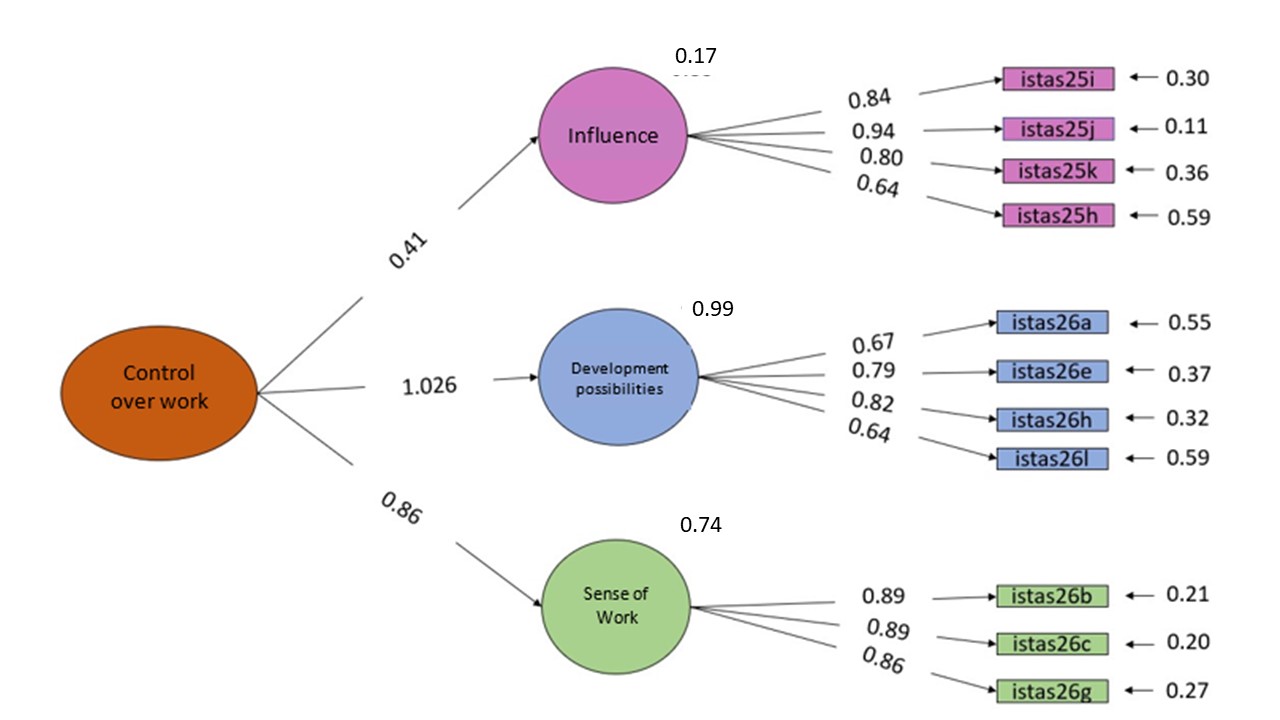


Supplementary figure 4. Social support and leadership quality dimension for the medium version.


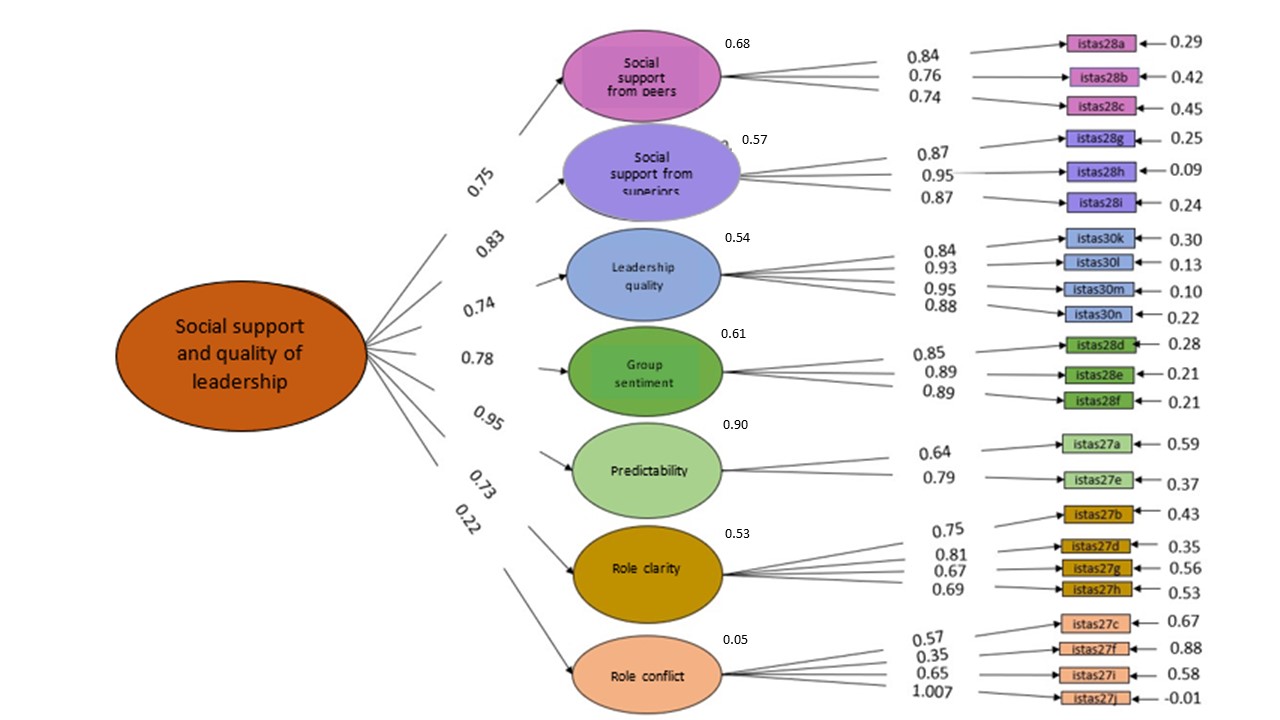


Supplementary figure 5. Work compensation dimension for the medium version.


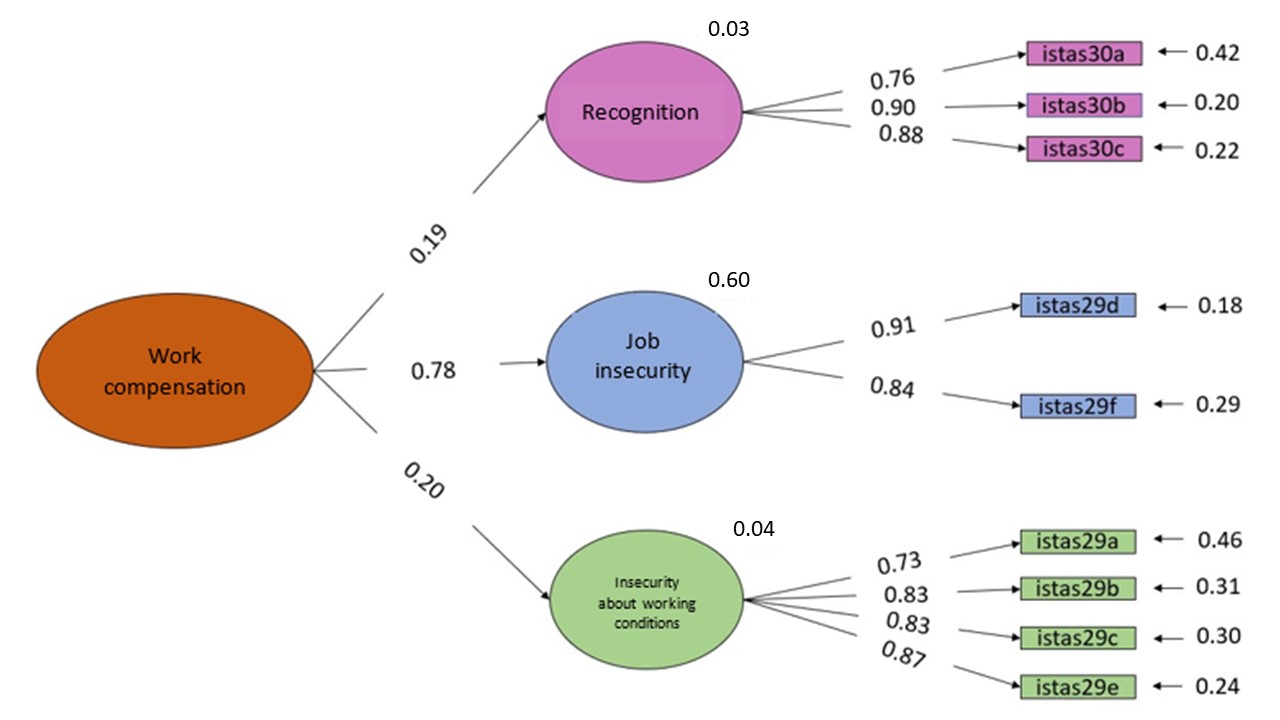


Supplementary figure 6. Capital stock dimension for the medium version.


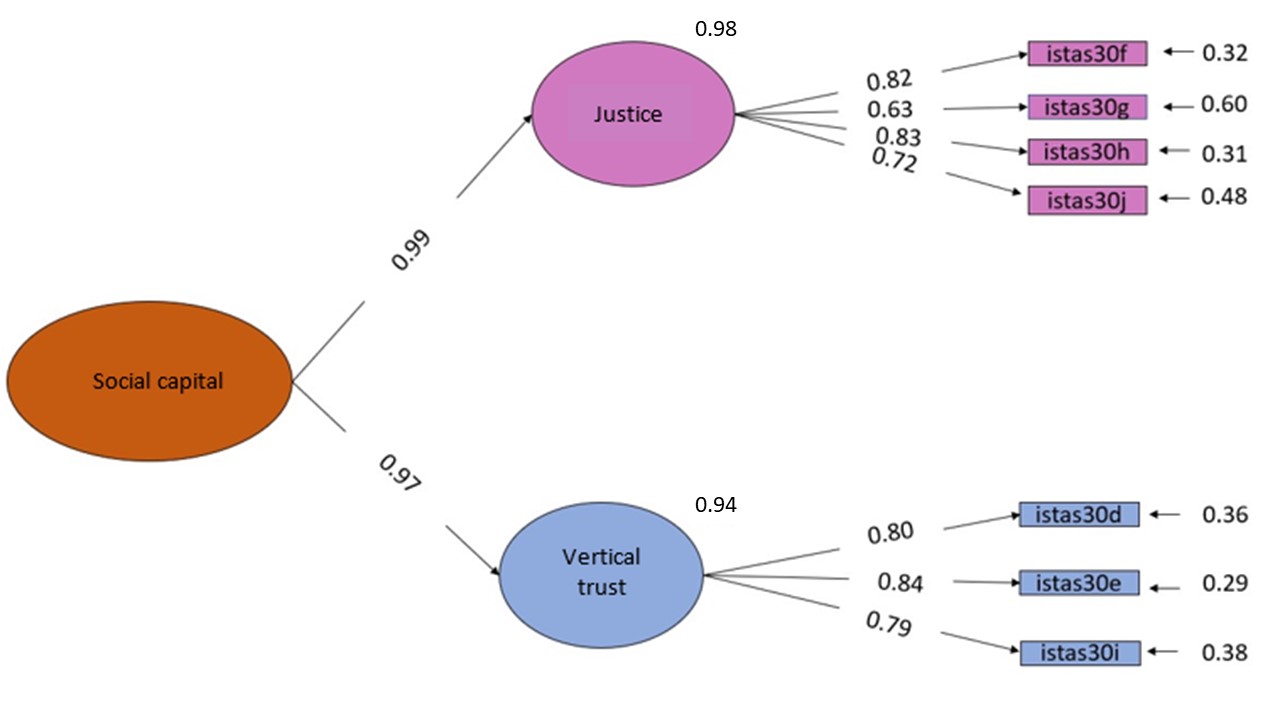


Supplementary figure 7. Family work conflict dimension for the short version.


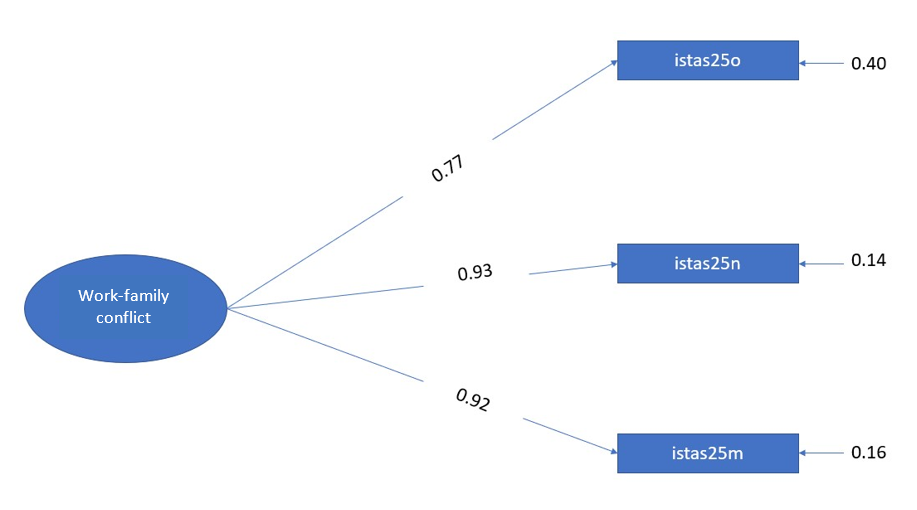


Supplementary figure 8. Dimension Control over work for the short version.


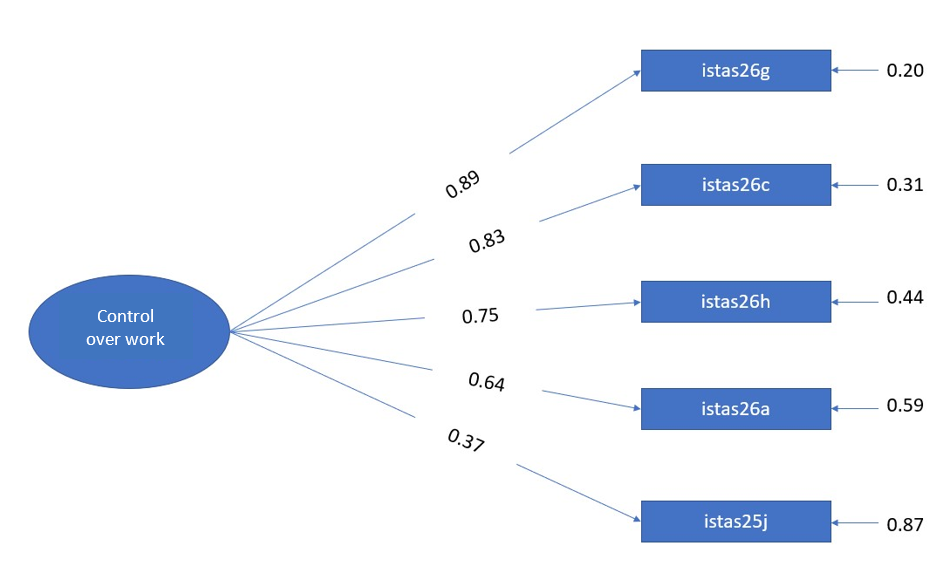
 Supplementary figure 9. Social support and leadership quality dimension for the short version.


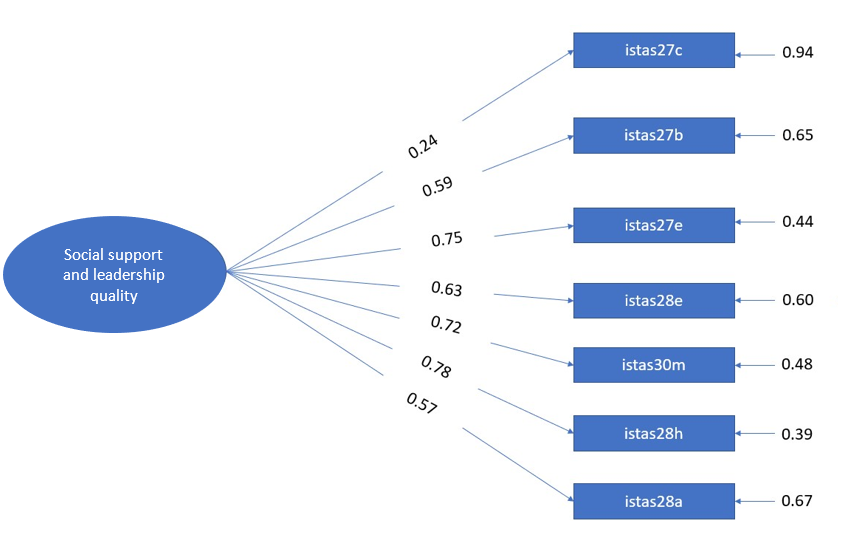


Supplementary figure 10. Work compensation dimension for the short version.


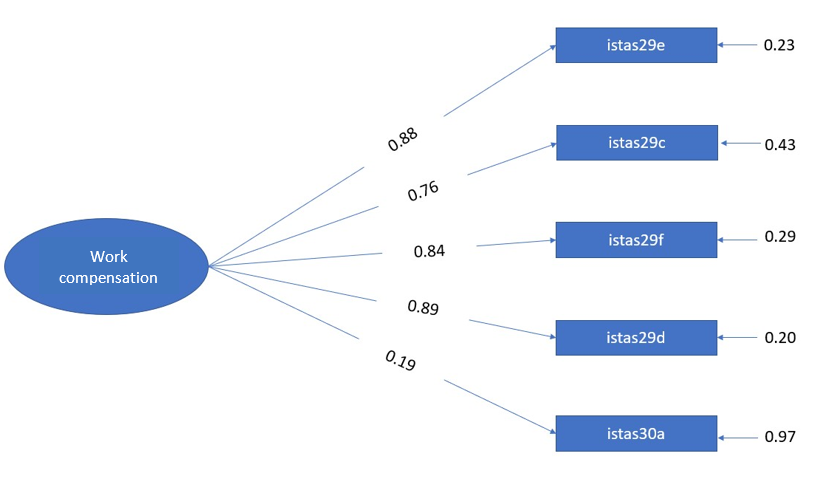


Supplementary figure 11. Capital stock dimension for the short version.


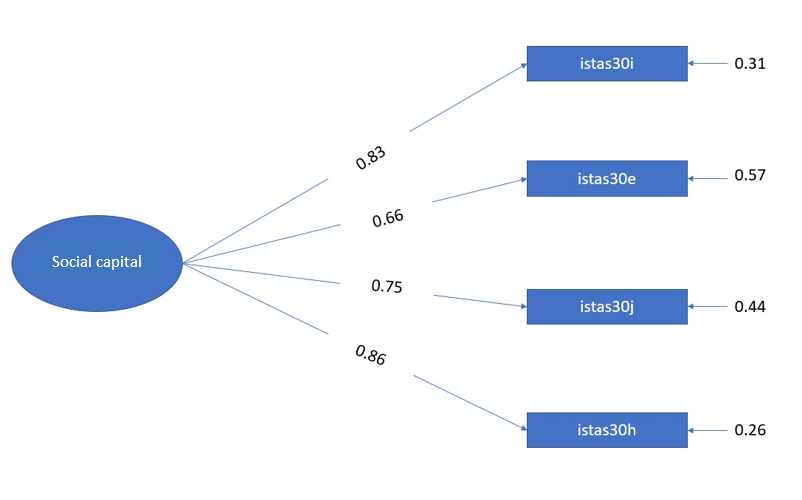


Supplementary figure 12. Dimension Psychological demands at work for the short version.


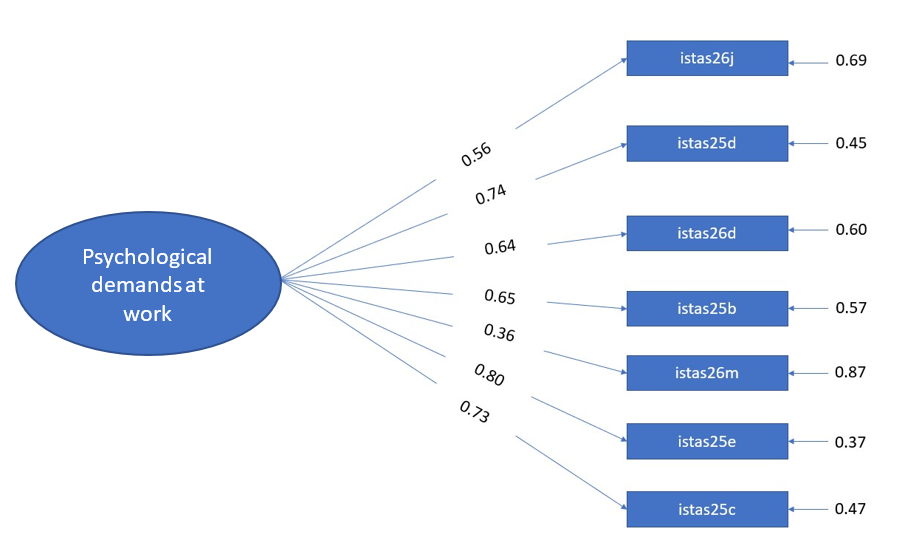


Supplementary table 1. Elimination of each of the possible models of the short version.

| Dimensions | Reduced version | *X^2^* | *df* | CFI | SRMR | RMSEA | α | Ω | Items removed |
| --- | --- | --- | --- | --- | --- | --- | --- | --- | --- |
| Psychological demands at work  (with 15 items in the original version) | 15 | 3600.6* | 90 | 0.792 | 0.098 | 0.151 [0.147-0.156] | 0.84 | 0.84 | - |
|  | 14 | 2391.3* | 77 | 0.858 | 0.082 | 0.133 [0.128-0.138] | 0.84 | 0.84 | 26f |
|  | 13 | 1624.5* | 65 | 0.901 | 0.069 | 0.111 [0.114-0.124] | 0.84 | 0.85 | 26f, 25f |
|  | 12 | 1291.7* | 54 | 0.912 | 0.067 | 0.116 [0.111-0.122] | 0.83 | 0.84 | 26f, 25f, 25q |
|  | 11 | 1057.1* | 44 | 0.914 | 0.066 | 0.116 [0.111-0.122] | 0.82 | 0.82 | 26f, 25f, 25q, 25p |
|  | 10 | 801.8* | 35 | 0.931 | 0.058 | 0.113 [0.107-0.120] | 0.82 | 0.83 | 26f, 25f, 25q, 25p, 26k |
|  | 9 | 459.0* | 27 | 0.950 | 0.051 | 0.097 [0.089-0.105] | 0.79 | 0.80 | 26f, 25f, 25q, 25p, 26k, 26i |
|  | 8 | 205.5* | 20 | 0.975 | 0.040 | 0.074 [0.065-0.083] | 0.78 | 0.79 | 26f, 25f, 25q, 25p, 26k, 26i, 25a |
|  | 7 | 184.8* | 14 | 0.975 | 0.040 | 0.085 [0.074-0.096] | 0.79 | 0.80 | 26f, 25f, 25q, 25p, 26k, 26i, 25a, 25g |
| Work-family conflict  (with 4 items in the original version) | 4 | 121.3* | 2 | 0.993 | 0.027 | 0.187 [0.160-0.216] | 0.88 | 0.88 | - |
|  | 3 | 0 | 0 | 1 | 0 | 0.000 [0.000-0.000] | 0.87 | 0.87 | 25l |
| Control overwork  (with 11 items in the original version) | 11 | 4986.3* | 44 | 0.820 | 0.204 | 0.257 [0.251-0.263] | 0.84 | 0.83 | - |
|  | 10 | 3860.1* | 35 | 0.834 | 0.174 | 0.253 [0.246-0.260] | 0.84 | 0.83 | 25k |
|  | 9 | 1612.1* | 27 | 0.915 | 0.081 | 0.186 [0.178-0.193] | 0.84 | 0.83 | 25k, 25i |
|  | 8 | 610.2* | 20 | 0.967 | 0.049 | 0.132 [0.123-0.141] | 0.84 | 0.84 | 25k, 25i, 25h |
|  | 7 | 307.6* | 14 | 0.981 | 0.042 | 0.111 [0.100-0.122] | 0.81 | 0.82 | 25k, 25i, 25h, 26e |
|  | 6 | 111.8* | 9 | 0.987 | 0.032 | 0.082 [0.069-0.096] | 0.77 | 0.77 | 25k, 25i, 25h, 26e, 26b |
|  | 5 | 56.0* | 5 | 0.993 | 0.026 | 0.077 [0.060-0.096] | 0.73 | 0.74 | 25k, 25i, 25h, 26e, 26b, 26l |
| Social support and quality of leadership  (with 23 items in the original version) | 23 | 15180.4* | 230 | 0.793 | 0.131 | 0.195 [0.193-0.198] | 0.89 | 0.89 | - |
|  | 22 | 12216.8* | 209 | 0.831 | 0.126 | 0.184 [0.181-0.186] | 0.89 | 0.89 | 27i |
|  | 21 | 10742.5* | 189 | 0.804 | 0.128 | 0.191 [0.188-0.194] | 0.90 | 0.90 | 27i, 27f |
|  | 20 | 11154.4* | 170 | 0.841 | 0.128 | 0.195 [0.192-0.198] | 0.91 | 0.91 | 27i, 27f, 27j |
|  | 19 | 10058.9* | 152 | 0.854 | 0.128 | 0.196 [0.192-0.199] | 0.91 | 0.91 | 27i, 27f, 27j, 27g |
|  | 18 | 9525.6* | 135 | 0.857 | 0.13 | 0.202 [0.199-0.206] | 0.91 | 0.91 | 27i, 27f, 27j, 27g, 27h |
|  | 17 | 8724.5* | 119 | 0.865 | 0.13 | 0.206 [0.202-0.210] | 0.90 | 0.90 | 27i, 27f, 27j, 27g, 27h, 27d |
|  | 16 | 7795.5* | 104 | 0.878 | 0.126 | 0.208 [0.204-0.212] | 0.90 | 0.90 | 27i, 27f, 27j, 27g, 27h, 27d, 28c |
|  | 15 | 7128.0* | 90 | 0.886 | 0.127 | 0.214 [0.210-0.219] | 0.90 | 0.90 | 27i, 27f, 27j, 27g, 27h, 27d, 28c, 28b |
|  | 14 | 6724.3* | 77 | 0.891 | 0.133 | 0.225 [0.221-0.230] | 0.89 | 0.89 | 27i, 27f, 27j, 27g, 27h, 27d, 28c, 28b, 27a |
|  | 13 | 5768.7* | 65 | 0.902 | 0.12 | 0.227 [0.222-0.232] | 0.89 | 0.89 | 27i, 27f, 27j, 27g, 27h, 27d, 28c, 28b, 27a, 28d |
|  | 12 | 3572.8* | 54 | 0.937 | 0.094 | 0.196 [0.190-0.201] | 0.88 | 0.88 | 27i, 27f, 27j, 27g, 27h, 27d, 28c, 28b, 27a, 28d, 28f |
|  | 11 | 3253.8* | 44 | 0.938 | 0.099 | 0.207 [0.201-0.213] | 0.87 | 0.87 | 27i, 27f, 27j, 27g, 27h, 27d, 28c, 28b, 27a, 28d, 28f, 30k |
|  | 10 | 2623.5* | 35 | 0.944 | 0.093 | 0.208 [0.202-0.215] | 0.84 | 0.84 | 27i, 27f, 27j, 27g, 27h, 27d, 28c, 28b, 27a, 28d, 28f, 30k, 28g |
|  | 9 | 1404.6* | 27 | 0.967 | 0.083 | 0.173 [0.165-0.181] | 0.82 | 0.82 | 27i, 27f, 27j, 27g, 27h, 27d, 28c, 28b, 27a, 28d, 28f, 30k, 28g, 28i |
|  | 8 | 1444.5* | 20 | 0.963 | 0.085 | 0.182 [0.173-0.191] | 0.79 | 0.77 | 27i, 27f, 27j, 27g, 27h, 27d, 28c, 28b, 27a, 28d, 28f, 30k, 28g, 28i, 30n |
|  | 7 | 287.9* | 14 | 0.962 | 0.046 | 0.107 [0.097-0.118] | 0.74 | 0.75 | 27i, 27f, 27j, 27g, 27h, 27d, 28c, 28b, 27a, 28d, 28f, 30k, 28g, 28i, 30n, 30l |
| Work compensation  (with 9 items in the original version) | 9 | 5170.5* | 27 | 0.799 | 0.275 | 0.334 [0.327-0.342] | 0.83 | 0.85 | - |
|  | 8 | 3877.5* | 20 | 0.818 | 0.143 | 0.336 [0.328-0.345] | 0.84 | 0.86 | 30c |
|  | 7 | 552.9* | 14 | 0.973 | 0.048 | 0.150 [0.140-0.161] | 0.86 | 0.88 | 30c, 30b |
|  | 6 | 341.5* | 9 | 0.981 | 0.039 | 0.147 [0.134-0.161] | 0.85 | 0.87 | 30c, 30b, 29a |
|  | 5 | 24.5* | 5 | 0.999 | 0.018 | 0.048 [0.030-0.068] | 0.81 | 0.85 | 30c, 30b, 29a, 29b |
| Social capital  (with 7 items in the original version) | 7 | 1282.5* | 14 | 0.929 | 0.077 | 0.231 [0.220-0.241] | 0.87 | 0.87 | - |
|  | 6 | 849.9* | 9 | 0.948 | 0.068 | 0.234 [0.221-0.247] | 0.87 | 0.87 | 30g |
|  | 5 | 540.4* | 5 | 0.954 | 0.059 | 0.251 [0.233-0.269] | 0.85 | 0.85 | 30g, 30d |
|  | 4 | 16.1* | 2 | 0.998 | 0.011 | 0.064 [0.038-0.095] | 0.81 | 0.82 | 30g, 30d, 30f |

*Notes:* Medium version = 69 items. Short version = 31 items. * Values are significant (p<0.05). X^2^ = Chi-squared. df = Degrees of freedom. CFI = Comparative fit index. RMSEA = Root mean square error of approximation. SRMR = Standardized root mean square residual. α =alpha coefficient of internal consistency. ω = omega coefficient of internal consistency.

Supplementary table 2. Items in English from CENSOPAS-COPSOQ.

| Item in English | Medium-version | Short-version |
| --- | --- | --- |
| 25a) Do you have to work very fast? | X |  |
| 25b) Do emotionally draining moments or situations occur in your work? | X | X |
| 25c) Are you late in delivering your work? | X | X |
| 25d) Does your job require you to keep your opinion to yourself? | X | X |
| 25e) Is the distribution of tasks irregular and causes you to accumulate work? | X | X |
| 25f) Does your job require you to treat everyone equally, even if you don't feel like it? | X |  |
| 25g) Do you have enough time to do your work? | X |  |
| 25h) Do you decide on the pace at which you work? | X |  |
| 25i) Do you influence decisions about your work? | X |  |
| 25j) Do you influence the way you do your work? | X | X |
| 25k) Do you influence what you do at work? | X |  |
| 25l) Are there times when you need to be at work and home at the same time? | X |  |
| 25m) Do you feel that your work generates so much fatigue that it impairs your home and family activities? | X | X |
| 25n) Do you feel that your work takes up so much of your time that it is detrimental to your domestic and family tasks? | X | X |
| 25o) Do you think about your household and family chores when you are working? | X | X |
| 25p) Do you find it impossible to finish your work activities? | X |  |
| 25q) In your work do you have to worry about or pay attention to other people's personal problems? | X |  |
| 26a) Does your work require you to take initiative? | X | X |
| 26b) Do the tasks you do make sense to you? | X |  |
| 26c) Do the tasks you do seem important to you? | X | X |
| 26d) Does your work affect you emotionally (in a negative way)? | X | X |
| 26e) Does your work allow you to learn new things? | X |  |
| 26f) Do you have to maintain a high work pace during your workday? | X |  |
| 26g) Do you feel committed to your work? | X | X |
| 26h) Does your job allow you to improve your knowledge and skills? | X | X |
| 26i) Is your job emotionally draining? | X |  |
| 26j) Does your job require you to guard your emotions and feelings? | X | X |
| 26k) Does your job require you to be kind to all people no matter how they treat you? | X |  |
| 26l) Does your job allow you to apply your skills, abilities, and knowledge? | X |  |
| 26m) Is the pace of your work high throughout the day? | X | X |
| 27a) In your job, are you informed well in advance of changes, important decisions and future projects? | X |  |
| 27b) Does your job have clear objectives? | X | X |
| 27c) Are there conflicting demands placed on you at work? | X | X |
| 27d) Do you know exactly which tasks are your responsibility? | X |  |
| 27e) Do you get all the information you need to do your job well? | X | X |
| 27f) Do you do things at work that are accepted by some people and not by others? | X |  |
| 27g) Do you know exactly what is expected of you at work? | X |  |
| 27h) Do you know exactly how much autonomy you have in doing your job? | X |  |
| 27i) Do you have to do tasks that you think should be done differently? | X |  |
| 27j) Do you have to perform tasks that seem unnecessary to you? | X |  |
| 28a) Do you receive help and support from your colleagues in carrying out your work? | X | X |
| 28b) Are your coworkers willing to listen to your work problems? | X |  |
| 28c) Do your coworkers talk to you about how you do your job? | X |  |
| 28d) Do you have a good working environment with your coworkers? | X |  |
| 28e) In your work do you feel that you are part of a workgroup or team? | X | X |
| 28f) Do you help each other at work? | X |  |
| 28g) Is your immediate boss willing to listen to your problems at work? | X |  |
| 28h) Do you receive help and support from your immediate boss in the performance of your work? | X | X |
| 28i) Does your immediate boss talk to you about how you do your job? | X |  |
| 29a) Are you worried about being transferred to another work center, area, department or section against your will? | X |  |
| 29b) Are you worried about having your schedule changed (shift, days of the week, start and end times, etc.) against your will? | X |  |
| 29c) Are you worried about having your tasks changed against your will? | X | X |
| 29d) Are you worried about being fired or not having your contract renewed? | X | X |
| 29e) Are you worried about having your salary or pay changed (not updated, lowered, the introduction of variable pays, payment in kind, etc.)? | X | X |
| 29f) Are you worried about how difficult it would be to find another job if you were to lose your job? | X | X |
| 30a) Is your work valued by management, management or leadership? | X | X |
| 30b) Is your work respected by management, the management or the boss? | X |  |
| 30c) Do you receive fair treatment from management, management or boss? | X |  |
| 30d) Does management have confidence that your employees are doing their jobs well? | X |  |
| 30e) Can you trust the information that comes from management, the management or the head? | X | X |
| 30f) Are conflicts resolved in a fair manner? | X |  |
| 30g) Are you recognized for a job well done? | X |  |
| 30h) Does management, the board of directors or the chief take proposals from all employees equally seriously? | X | X |
| 30i) Are workers allowed to express their opinions and emotions? | X | X |
| 30j) Are tasks distributed in a fair way? | X | X |
| 30k) Does your current line manager make sure that every employee has good opportunities for career development? | X |  |
| 30l) Does your current immediate supervisor plan the work well? | X |  |
| 30m) Does your current immediate supervisor distribute the work well? | X | X |
| 30n) Does your current immediate supervisor resolve labor conflicts well? | X |  |

Supplementary table 3. Correlation between the dimensions of the short version (columns) with the second-order dimensions and subdimensions of the medium version (rows).

|  |  | Dimensions in short version | | | | | |
| --- | --- | --- | --- | --- | --- | --- | --- |
| N° | Dimensions and Sub-dimensions in medium version | 1 | 2 | 3 | 4 | 5 | 6 |
| 1 | Psychological demands at work | **0,92** | 0,61 | 0,19 | 0,24 | 0,08 | 0,13 |
| 2 | Work-family conflict | 0,56 | **0,97** | 0,30 | 0,32 | 0,21 | 0,16 |
| 3 | Control over work | 0,23 | 0,27 | **0,91** | 0,50 | 0,15 | 0,40 |
| 4 | Social support and quality of leadership | 0,34 | 0,39 | 0,52 | **0,93** | 0,30 | 0,68 |
| 5 | Work compensation | 0,15 | 0,27 | 0,24 | 0,39 | **0,95** | 0,41 |
| 6 | Social capital | 0,22 | 0,17 | 0,39 | 0,64 | 0,29 | **0,96** |
| 7 | Quantitative requirements | *0,81* | 0,56 | 0,34 | 0,36 | 0,09 | 0,22 |
| 8 | Pace of work | *0,46* | 0,15 | -0,25 | -0,16 | -0,14 | -0,16 |
| 9 | Emotional demands | *0,80* | 0,67 | 0,22 | 0,25 | 0,12 | 0,14 |
| 10 | Demands to hide emotions | *0,66* | 0,36 | 0,12 | 0,09 | 0,05 | 0,06 |
| 11 | Double presence | 0,56 | *0,97* | 0,30 | 0,32 | 0,21 | 0,16 |
| 12 | Influence | 0,10 | 0,04* | *0,53* | 0,21 | 0,04* | 0,21 |
| 13 | Development possibilities | 0,18 | 0,34 | *0,83* | 0,54 | 0,22 | 0,41 |
| 14 | Sense of work | 0,30 | 0,35 | *0,77* | 0,51 | 0,10 | 0,37 |
| 15 | Social support from peers | 0,11 | 0,23 | 0,34 | *0,60* | 0,21 | 0,39 |
| 16 | Social support from superiors | 0,08 | 0,19 | 0,34 | *0,73* | 0,26 | 0,57 |
| 17 | Leadership quality | 0,19 | 0,16 | 0,33 | *0,69* | 0,18 | 0,70 |
| 18 | Group sentiment | 0,22 | 0,26 | 0,43 | *0,70* | 0,19 | 0,37 |
| 19 | Predictability | 0,19 | 0,24 | 0,33 | *0,69* | 0,30 | 0,53 |
| 20 | Role clarity | 0,22 | 0,30 | 0,58 | *0,65* | 0,20 | 0,45 |
| 21 | Role conflict | 0,59 | 0,41 | 0,22 | *0,37* | 0,12 | 0,24 |
| 22 | Recognition | 0,15 | 0,18 | 0,37 | 0,62 | *0,32* | 0,68 |
| 23 | Job insecurity | 0,06 | 0,19 | 0,11 | 0,15 | *0,92* | 0,14 |
| 24 | Insecurity about working conditions | 0,13 | 0,24 | 0,12 | 0,21 | *0,88* | 0,19 |
| 25 | Justice | 0,25 | 0,17 | 0,34 | 0,58 | 0,26 | *0,90* |
| 26 | Vertical trust | 0,17 | 0,15 | 0,41 | 0,63 | 0,27 | *0,88* |

Note: All values are significant (p<0.05), except those with an asterisk (*). Values in bold are the correlation values between the medium and short version. Values in italics represent the relationship between the unidimensional scales and the short version.
